# The Association of Divorce and Late-Life Brain Health in a Racially and Ethnically Diverse Cohort of Older Adults

**DOI:** 10.1093/geroni/igaf122.4023

**Published:** 2025-12-31

**Authors:** Suhani Amin, Junxian Liu, Paola Gilsanz, Rachel Whitmer, Charles DeCarli, Eleanor Hayes-Larson

**Affiliations:** University of Southern California, Los Angeles, California, United States; University of Southern California, Los Angeles, California, United States; Kaiser Permanente Northern California, Pleasanton, California, United States; University of California, Davis, Sacramento, California, United States; University of California, Davis, Sacramento, California, United States; University of Southern California, Los Angeles, California, United States

## Abstract

Divorce is a common life stressor that could impact brain health in older adults. Current literature evaluating its relationship with neuroimaging measures, including structural MRI and amyloid PET, is limited and contains mixed findings. Using the Kaiser Healthy Aging and Different Life Experiences (KHANDLE) and Study of Healthy Aging in African Americans (STAR) datasets, we analyzed the impact of history of divorce on MRI outcomes including volumetric measures and white matter hyperintensities (n = 649) using linear regression models and on PET outcomes (n = 374), including standardized uptake value ratio using linear regression and amyloid positivity using relative risk regression. All models adjusted for age at brain imaging, gender, race/ethnicity, southern birth, participant’s education, parental separation or divorce, parental education, and childhood socioeconomic status. The sample had a mean age at baseline of 72.4 years (SD = 7.8), with 60.7% of participants female and 42.5% of the sample reporting a history of divorce. Those with a history of divorce had smaller hippocampus volume, greater total white matter hyperintensity volume, and increased amyloid burden, but effect estimates were small, imprecise, and crossed the null. Results were similar when stratified by sex and restricted to individuals age 65+. Thus, a history of divorce was not strongly associated with late-life brain health in our sample, and differences may reflect social and economic contexts surrounding partnership dissolution. Further research should investigate possible sources of bias and heterogeneity, such as differences in the subjective experience of divorce as a stressor, in understanding this relationship.

